# Depressive Symptoms and Psychosocial Functioning in Preadolescent Children

**DOI:** 10.1155/2011/548034

**Published:** 2011-03-30

**Authors:** Marita McCabe, Lina Ricciardelli, Sophie Banfield

**Affiliations:** School of Psychology, Deakin University, 221 Burwood Highway, Burwood, VIC 3125, Australia

## Abstract

The current study was designed to determine the percentage of children “at-risk” of depression or evidencing clinical levels of depression. In addition, the study examined how the “at-risk” and the clinical groups differed from children who demonstrated no depressive symptoms on positive and negative affect, four aspects of self-concept, and peer ratings of popularity. Respondents were 510 children (270 boys 240 girls) who ranged in age from 7 to 13 years (mean = 9.39). The results demonstrated that 23% of children were either in the “at-risk” or clinical range of depression. Children in both the clinical and the “at-risk” range demonstrated higher negative affect but lower positive affect and lower self-concepts than children in the normal range. However, children's peers only differentiated between the “clinical” and “normal” groups. It is harder for peers, and other informants such as teachers and parents, to detect the problems of children with elevated depressive symptoms but who do not meet the diagnostic criteria. It is important to implement intervention programs for children who evidence depression symptoms, as well as “at-risk” children. “At-risk” children with elevated levels of depressive symptoms may be more disadvantaged, as their problems are less likely to be detected and treated.

## 1. Introduction

The clinical presentation of depressive symptoms in children largely parallels that of adults [1, 2]. However, as outlined below, there are some differences in the presentation of these symptoms across the life span [3]. The diagnostic and statistical manual of mental disorders [4] provides a summary of the most widely accepted constellation of depressive symptoms associated with each depressive disorder. The two most prevalent in childhood, and therefore most relevant to the current research, are major depressive disorder (mdd) and dysthymic disorder (DD) [4]. It is important to obtain a better understanding of the prevalence of depressive disorders in childhood, the prevalence of those at-risk of developing depression, and the factors in childhood that are associated with these depressive symptoms.

Epidemiological studies of community samples have reported the prevalence of MDD in children to range from 0.4–2.5%, while the prevalence of DD has been reported to range from 0.6–1.7% (e.g., [5–7]). However, the number of children exceeding cutoff scores for clinically significant levels of depressive symptoms as assessed by the children's depression inventory (CDI) has been shown to range from 20 to 24% [8, 9]. 

Symptoms associated with depression can cause significant impairment across emotional, physical, behavioral, cognitive, and interpersonal functioning [10, 11]. Poor peer relationships, low self-concept, and high negative affect have been strongly associated with depression in preadolescent children [12–14]. Depressed children have been found to demonstrate lower rates of prosocial behavior [15], have poor friendship quality [16], and tend to elicit negative reactions and rejection when interacting with peers [15]. Additionally, depressed children are reported to be sensitive to negative social cues, incorporating this feedback into their social perception [12]. Depression in children has also been associated with poor self-concept, with children tending to evaluate themselves negatively, to have low expectations for performance, more stringent criteria for failure, and a lower perceived self-competence [17]. Poor self-concept has also been correlated with a wide range of negative outcomes, including higher rates of suicide, loneliness, depression, social anxiety, and alienation in childhood and adolescence [18]. 

The above research has demonstrated an association between depression and poor interpersonal relationships, poor self-concept, high negative affect, and a lack of positive affect. However, a comparison of the psychosocial functioning of the “at-risk” group of preadolescents with elevated depressive symptoms to that of the normal and a clinical group has yet to be examined. This is important as researchers have argued that treatment may be appropriate for children that evidence functional impairment even though children may not meet diagnostic criteria for depression [11, 19]. These “at-risk” children with elevated levels of depressive symptoms may suffer continuing problems and may be more disadvantaged as their problems are less likely to be detected and treated. Also, given the imperfections of the DSM nosology [19], it is important to also consider those with impairing symptoms. For example, Gotlib et al. [11] has highlighted the importance of being clinically sensitive to adolescents who presented with elevated levels of depressive symptomatology, but who did not meet diagnostic criteria for a depressive disorder, as they reported marked difficulties in psychosocial functioning.

The current study examined the relationship between depressive symptoms and the above variables among preadolescent children (i.e., children aged between 8 and 11 years). Particular emphasis was placed on the examination of the psychosocial functioning of children who reported depressive symptoms in the normal, “at-risk” and clinical range of depressive symptoms. We firstly examined whether the level of depressive symptoms was similar in boys and girls and across year levels, and whether there would be any interaction between these two variables. That is, are there gender differences in levels of depression among children, and is the trajectory of change with increasing age different for boys and girls. These gender differences according to grade have yet to be fully evaluated in previous studies, and they have implications for the clinical management of depression among children. 

Further, it was hypothesised that negative affect, poor self-concept and poor peer popularity would become more severe as the level of depressive symptoms increased, with those in the clinical range demonstrating significantly more problems in their interpersonal relationships, self-concept and affect, compared to those “at-risk”, who would evidence more problems than those in the normal range. We also included a peer-report measure of peer acceptance/popularity, as the detection of depressive symptoms by peers is critical. Children with depression see themselves and their environment in a negative light. Peers detect this negativity and then dislike interacting with them [20].

## 2. Method

### 2.1. Participants

The 510 participants (270 boy, 240 girls) were enrolled in Grades 3 to 6 at six primary schools in urban regions in Melbourne, Australia. These schools included students from diverse socioeconomic and cultural backgrounds. The only demographic information gathered was on the child's sex, age in years, and grade level. There were 106 boys and 102 girls in grade 3 (*M* = 8.27 years, SD = 0.48), 67 boys and 48 girls in grade 4 (*M* = 9.32 years, SD = 0.50); 60 boys and 47 girls in grade 5 (10.13 years, SD = .40), and 60 boys and 43 girls in grade 6 (9.35 years, SD = 1.18).

### 2.2. Materials

#### 2.2.1. The Children's Depression Inventory (CDI)

The CDI is a 27-item self-report measure of severity of depressive symptoms in children as young as seven. The CDI is a childhood extension of the Beck depression inventory [21]. In the present study, one modification was made to the original CDI, which was the removal of the item that assesses suicidal ideation. This item was removed because of ethical considerations (the question was of concern to some of the schools) and this is in line with other previous studies [22–24]. Scores ranged from 0 to 52, with higher scores indicating severe levels of depressive symptoms and scores 12 or below indicating depressive symptoms in the normal range [25]. Since scores of greater than 19 were considered to be in the clinical range, participants who obtained scores of 13–19 were classified as being “at-risk” for depression. The CDI has demonstrated good validity, high internal consistency and test-retest reliability in the measurement of depressive symptoms [26]. The Cronbach's alpha coefficients in the current study were .89 for the total sample and for both boys and girls.

#### 2.2.2. Positive and Negative Affect Schedule for Children (PANAS-C)

The PANAS-C [27] assesses positive and negative affect in children. It is a 20-item self-report measure consisting of two scales: a 10-item positive affect scale and a 10-item negative affect scale. Scores on the PANAS-C range from 10 to 50 on each scale. High scores on the negative affect and positive affect scales indicate elevated level of negative affect and positive affect, respectively. Laurent et al. [27] reported high internal consistency, good construct validity, and convergent and discriminant validity for the PANAS-C with children aged between 8 and 18 years. The Cronbach's alpha coefficients in the current study were .73 for boys and .74 for girls for the positive affect scale, and .82 for boys and .85 for girls for the negative affect scale.

#### 2.2.3. Perceived Competence Scale for Children (PCSC)

The PCSC [28] is a self-report instrument that assesses a child's self-concept across four domains: academic (academic performance), social (confidence with and acceptance by peers), sporting (sporting and outdoor activities), and global self-worth (being sure of oneself and what one does). The PCSC contains seven items in each subscale, with a total of 28 items. Participants responded using a four-point Likert scale with possible responses of false, mostly false, mostly true and true. Scores are summed and averaged for each subscale, resulting in separate subscale means. High scores on each of these subscales indicated positive levels of self-concept. Harter [28] reported good internal consistency for each of the subscales (alphas ranging from .73 to .86) and satisfactory discriminant validity. The Cronbach's alpha coefficients in the current study for the academic (.73 for boys, .75 for girls), social (.79 for boys, .82 for girls), sporting (.73 for boys, .74 for girls) and global self-worth (.74 for boys, .76 for girls) domains indicated good reliability.

#### 2.2.4. Peer-Report Measure of Peer Acceptance/Popularity

The peer-report measure of peer acceptance/popularity [29] is a sociometric scale that requires each participant to nominate three classmates in response to the following two items: “you like to play with a lot” and “you like to play with the least”. However, because of the sensitivity of the second item it was altered to ask the participants to name three classmates “you like to play with a little”. For each child, the number of nominations he or she received on each of the two items were added and were standardised for differences in classroom size. This was achieved by dividing the number of nominations received by the number of students in the class in which the child was a member. The peer-report measure of peer acceptance/popularity has been found to have moderate to high levels of short-term stability and concurrent validity [30, 31]. Since there was no retest, and no other measure of peer popularity, it was not possible to calculate these measures in the current study.

#### 2.2.5. Procedure

Ethics Approval was obtained from the University Ethics Committee. An information pack was sent to 24 primary schools, which represented diverse sociocultural areas in Melbourne, Australia. The school principals from six schools agreed participate in the study. All children in the selected classes were invited to participate in the study (*N* = 794), but written parental consent was required for children to take part in the study. If parents wanted any further information they were asked to contact the researchers.

Parental consent was 67% (*N* = 532, class range 48% to 100%), with 96% of these participants completing the questionnaire. The anonymous questionnaire was completed in class groups of about 20 children. The researcher verbally presented the questionnaire to the class group and students were asked to answer items as they were read out. Children were encouraged to ask questions if they needed to clarify the meaning of an item or if they required assistance. In addition, if any children felt discomfort with any question then they were told that they could leave it out. The school counsellor was also advised about the study so that any child who was distressed in any way could speak to her/him and they were also given the contact number for Kids' Helpline in Australia, which provides a telephone service for children who may want to speak to a counsellor for any problems.

## 3. Results

The mean level of depressive symptoms reported by the overall sample of participants in the current study fell within the normal range (*M* = 8.65, SD = 7.56) [21]. These levels are comparable to those found in other studies that used the complete CDI scale (one item was removed in the current study) [25, 32, 33]. 

Table 1 displays the adjusted mean, standard error, significance level, and intraclass correlation coefficient (ICC) for the measures of depressive symptoms, affect, self-concept, and peer-rated popularity for the main effects of sex and grade, and the sex by grade interaction. There were no differences between grade 3 and 4, or between grade 5 and 6, thus these respective grades were combined. ICC is a measure of the extent to which observations are not independent of a grouping variable (e.g., schools). It is a ratio of variance between groups in the model to variance within these groups. The presence of a significant intraclass correlation is an indicator of the need to employ multilevel modeling. Higher percentages indicate that the grouping level makes a difference. As the ICC levels were low, a conventional analysis of variance was used. 

**Figure 1:**
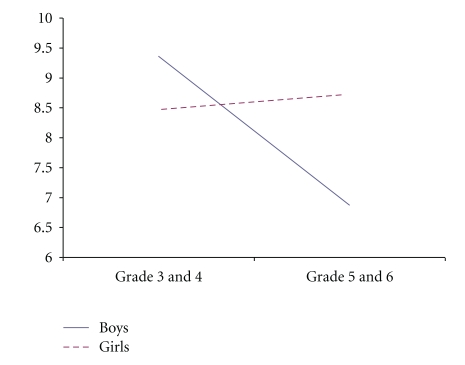
Sex by grade interaction for depressive symptoms.

**Figure 2:**
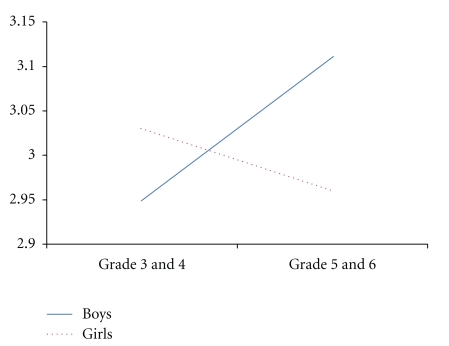
Sex by grade interaction for academic self-concept.

**Figure 3:**
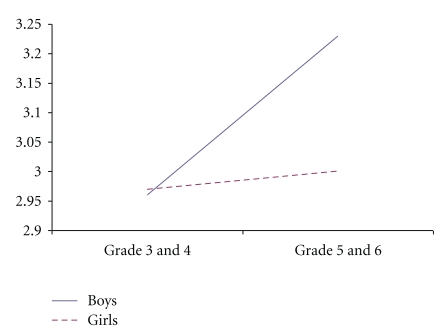
Sex by grade interaction for social self-concept.

**Figure 4:**
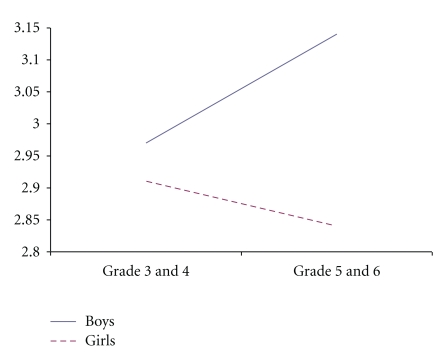
Sex by grade interaction for sporting self-concept.

The findings revealed that there were gender differences in sporting self-concept, with boys scoring higher than girls. Further, Grades 5 and 6 scored higher in sporting self-concept than Grades 3 and 4. These differences are further highlighted in the significant interactions summarized below. There was a significant sex by grade interaction depressive symptoms, and academic, social and sporting self-concepts (see Figures 1, 2, 3, and 4). Fisher's LSDs (*P* < .05) revealed that boys in Grades 5 and 6 reported significantly lower levels of depressive symptoms than boys in Grades 3 and 4 but higher academic, social and sporting self-concepts. The power for these analyses was low (.10 to .56), but this is not surprising given the small number of children in the “at-risk” and clinical groups.

The number and percentage of participants falling within the normal, “at-risk” and clinical range of depressive symptoms, and their means and standard deviations are presented by sex, grade and for the total sample in Table 2. Just over 77% of students in the sample reported little to no depressive symptoms. Nearly 13% of participants fell within the “at-risk” range for depression, while almost 10% reported depressive symptoms that were in the clinical range. 

A second analysis of variance was conducted to determine the relationship between depressive symptoms, positive and negative affect, the four aspects of self-concept and peer-rated popularity. The analyses revealed that as the level of depressive symptoms reported by students increased, levels of positive affect, academic self-concept, social self-concept, sporting self-concept, global self-worth and peer-rated popularity decreased, while levels of negative affect increased. Negative affect (.43), academic self-concept (−.32), social self-concept (−.39) and global self-worth (−.28) demonstrated the largest change per unit increase of depressive symptoms compared to positive affect (−.21) sporting self-concept (−.23) and peer-rated popularity (−.20), which demonstrated more modest changes (see Table 3).

Fisher's LSD tests indicated that individuals with depressive symptoms in the normal range reported significantly higher levels of positive affect, academic self-concept, social self-concept, sporting self-concept, global self-worth and lower levels of negative affect compared to individuals reporting depressive symptoms in the “at-risk” and clinical range at the *P* < .001 level of significance (see Table 3). Those individuals in the “at-risk” range also reported higher levels compared to those in the clinical range on academic self-concept, social self-concept and global self-worth at the *P* < .001 level, and positive affect and sporting self-concept at the *P* < .05 level, as well as lower levels of negative affect at the *P* < .001 significance level (see Table 3). Individuals falling within the normal range of depressive symptoms were rated by their peers as being more popular than children with clinical levels of depressive symptoms. The popularity of children within the “at-risk” range of depressive symptoms fell between those in the normal and clinical range, and did not differ significantly from either (see Table 3).

## 4. Discussion

Depressive symptoms reported by the overall sample in the current study fell within the normal range, and were similar for boys and girls and across year levels. However, there was a sex by grade interaction, with boys in Grades 5 and 6 reporting significantly lower levels of depressive symptoms than boys in Grades 3 and 4. Consistent with this finding, we also showed that boys in Grades 5 and 6 scored higher on academic, social and sporting self-concepts. It is now important to determine what other emotional, social and cognitive changes are occurring among boys in Grades 5 and 6 that may explain these findings. 

In total, 23% of children reported depressive symptoms in the “at-risk” and clinical range, and these levels did not differ across sex or grade. The above results are consistent with previous research with children [8, 9, 34], and adolescents [35]. When comparing the current findings to previous epidemiological studies of the prevalence of depressive disorders in community samples of children, it is evident that a much higher proportion of children are presenting with elevated depressive symptomatology than would meet the formal psychiatric diagnostic criteria for a depressive disorder [5–7, 11, 36]. Gotlib et al. [11] highlighted the importance of being clinically sensitive to adolescents who presented with elevated levels of depressive symptomatology. This study highlights the importance of also being clinically sensitive to preadolescents. 

Levels of psychosocial functioning were highest for children with depressive symptoms in the normal range, but generally deteriorated as the level of depressive symptoms became more severe. Consistent with the findings of Gotlib et al. [11], the current study found that children with “at-risk” and clinical levels of depression reported psychosocial disturbances, higher negative affect, and reduced positive affect and self-concept, when compared to children presenting with depressive symptoms in the normal range. An important aspect to the findings from the current study is that children who were classified as being “at-risk” of depression demonstrated problems in their psychosocial functioning, not just those students who were classified as being in the clinical range. These findings are consistent with past research [12–14]. If these symptoms remain in place into adolescence, it is likely that the poor self-concept associated with high levels of depressive symptoms may cause problems in other aspects of the children's lives. 

However, only children with clinical levels of depressive symptoms were rated by their peers as less popular that those who scored in the normal range. It may be that children in the “at-risk” group do not stand out sufficiently to be noticed by their peers. Teachers have also been found to be not able to detect less severe cases. Kleftaras and Didaskalou [34] found that although 30 percent of 5th and 6th grade children evidenced high levels of depressive symptoms, their teachers failed to identify them as being depressed, and attributed behavioral problems to other causes. Thus, many children “at-risk” may be unlikely to obtain appropriate intervention for their depression. These issues now need to be explored further in longitudinal research. 

Harrington et al. [37] assessed the continuity of childhood depressive symptoms into adulthood. They found that depressed children and adolescents were at greater risk of developing a depressive disorder in adult life as well as having an increased risk for psychiatric hospitalisation and psychiatric treatment. This indicates that there may be a continuity of affective disturbance between childhood and adult life. Weissman et al. [38] also investigated the continuity of prepubertal major depressive disorder into adulthood and reported similar results to those of Harrington et al. [37]. These results highlight the importance of addressing depressive symptoms in childhood in order to prevent the continuation of these symptoms into adulthood, with the associated risk of the symptoms developing into MDD.

A limitation of the current study was that it was cross-sectional in nature, and so it was not possible to determine the direction of the relationships between the variables. It is important that the participants are followed up over time in order to determine the extent to which the depressive symptoms continue into adolescence, and also to determine the directional relationships between the variables. While schools were selected to represent diverse sociocultural areas in Melbourne, Australia, we did not collect specific data on socioeconomic background and we were also not able to assess how the ones who participated in this study differed from those who did not. Another limitation is that the number of children in the clinical group was small so the findings need to be verified with a larger sample. It is also possible that the level of risk of clinical depression may have been underestimated due to the removal of the item in the CDI that related to suicidal ideation. The study also relied heavily on self-reports of depressive symptoms and psychosocial functioning. Obtaining information on how others, including parents and teachers, provides additional information on children's functioning and symptoms [39–41]. Future studies also need to examine the role of other factors such socioeconomic status, ethnicity, family situations, and academic performance.

In summary, the results suggest that it is important to consider interventions for both “at-risk” and clinically depressed children in order to improve their mood, self-concept and social function, and so attempt to prevent the development of clinical depression in adolescence and adulthood. “At-risk” children with elevated levels of depressive symptoms also demonstrated higher negative affect but lower positive affect and lower self-concepts than children with depressive symptoms in the normal range.

## Figures and Tables

**Table 1:** Mean scores and main effects of sex and grade and the sex by grade interaction for depressive symptoms, affect, and self-concept.

Outcome	Depressive symptoms	Positive affect	Negative affect	Academic self-concept	Social self-concept	Sporting self-concept	Global self- worth
Sex							
Boys	8.11	38.79	21.31	3.03	3.09	3.06	3.25
Girls	8.59	38.18	22.39	3.00	2.99	2.88	3.24
Standard error	.69	.50	.57	.05	.06	.06	.05
*P*	.87	.09	.15	1.00	.19	.00	.86
Grade							
Grades 3 & 4	8.92	38.77	22.61	2.99	2.97	2.94	3.23
Grades 5 & 6	7.79	38.20	21.09	3.04	3.11	2.99	3.26
Standard error	.94	.55	1.05	0.07	.08	.06	.05
*P*	.22	.25	.08	.43	.05	.33	.48
Sex by grade							
Boys Grades 3 and 4	9.36	39.49	22.27	2.95	2.96	2.97	3.23
Boys Grades 5 and 6	6.87	38.10	20.36	3.11	3.23	3.14	3.27
Girls Grades 3 and 4	8.47	38.06	22.95	3.03	2.97	2.91	3.29
Girls Grades 5 and 6	8.72	38.29	21.83	2.96	3.00	2.84	3.19
Standard error	1.18	.80	1.20	.09	1.00	.09	.07
*P*	.05	.11	.48	.03	.05	.03	.12
ICC	4.64%	1.06%	11.43%	3.60%	4.18%	2.91%	0.56%

**Table 2:** Number of participants falling within the normal, “at-risk”, and clinical range of depressive symptoms and the mean level of depressive symptoms across sex and grade.

		Normal (CDI < 13)	“At risk” (CDI 13–19)	Clinical (CDI > 19)
Sex				
Boys	*M* (SD) *n* %	5.21 (3.45) 205 75.9	15.52 (1.99) 42 15.6	26.48 (5.54) 23 8.5
Girls	*M* (SD) *n* %	5.49 (3.35) 190 79.2	15.21 (1.82) 24 10.0	26.04 (6.73) 26 10.8
Grade				
Grades 3 and 4	*M* (SD) *n* %	5.51 (3.42) 247 76.5	15.51 (1.84) 45 13.9	26.87 (6.90) 31 9.6
Grades 5 and 6	*M* (SD) *n* %	5.02 (3.37) 148 79.1	15.19 (2.11) 21 11.2	25.17 (4.53) 18 9.6

Total	*M* (SD) *n* %	5.35 (3.40) 395 77.5	15.41 (1.92) 66 12.9	26.24 (6.14) 49 9.6

**Table 3:** Main effect of depressive symptom level on affect, self-concept, and peer-rated popularity.

	Normal (CDI < 13)	“At risk” (CDI 13–19)	Clinical (CDI >19)	ICC	Standard error	*P*	Normal versus “at-risk” *t*	Normal versus clinical *t*	“At risk” versus clinical *t*
Positive affect	39.35	36.88	34.77	1.48%	1.00	<.001	3.48***	5.65***	2.11*
Negative affect	20.83	24.11	29.46	13.3%	1.06	<.001	4.29***	10.03***	5.05***
Academic self-concept	3.14	2.65	2.28	3.00%	.67	<.001	7.29***	11.28***	3.87***
Social self-concept	3.20	2.52	2.10	3.98%	.73	<.001	9.27***	13.03***	3.93***
Sporting self-concept	3.08	2.69	2.42	1.03%	.76	<.001	5.02***	7.49***	2.45*
Global self-concept	3.39	2.96	2.54	0.71%	.56	<.001	7.62***	13.20***	5.30***
Peer-rated popularity	.21	.19	.18	30.75%	.02	.05	1.49	2.14*	0.66

*P < .05; ***P < .001.
